# Supplementary material for: One-second coherence for a single electron spin coupled to a multi-qubit nuclear-spin environment
Source: Nat Commun. 2018 Jun 29;9:2552. doi: 10.1038/s41467-018-04916-z (PMC6026183; doi:10.1038/s41467-018-04916-z)
Supplement: Supplementary file 1 — Supplementary Information [file 41467_2018_4916_MOESM1_ESM.pdf]

Supplementary Information for "One-second coherence for a single electron spin coupled to a multi-qubit nuclear-spin environment"

Abobeih et al.

Supplementary Note 1: System Hamiltonian.

The Hamiltonian describing a system composed of an NV center and a ^{13}C nuclear spin environment, in a suitable rotating frame and under the secular approximation can be described by:

$$\hat{H} = \sum_{i=1}^{n} (\omega_0 \hat{I}_z^i + A_{\parallel}^i \hat{S}_z \hat{I}_z^i + A_{\perp}^i \hat{S}_z \hat{I}_x^i) + \hat{H}_{n-n}, \quad (1)$$

where ω_o $(= 2\pi \cdot \gamma_c B_z)$ is the Larmor frequency, $A_{\parallel}(A_{\perp})$ is the parallel (perpendicular) hyperfine coupling between the electron and ^{13}C nuclear spin with respect to the applied static magnetic field. The dipolar interaction between ^{13}C nuclear spins in the environment H_{n-n} is given by:

$$\hat{H}_{n-n} = \sum_{i>j} \frac{\mu_o}{4\pi} \frac{\gamma_c^i \gamma_c^j}{r_{ij}^3} \left[\mathbf{I}^i \cdot \mathbf{I}^j - \frac{3(\mathbf{I}^i \cdot \mathbf{r}_{ij})(\mathbf{I}^j \cdot \mathbf{r}_{ij})}{r_{ij}^2} \right], \quad (2)$$

where γ_c is the gyromagnetic ratio of the nuclear spin, $\mathbf{r}_{ij}$ is the vector connecting the two nuclear spins and $\mathbf{I}^i$ is the spin-$\frac{1}{2}$ operator. Now we can rewrite the Hamiltonian as follows:

$$\hat{H} = |0\rangle \langle 0| \hat{H}_0 + |1\rangle \langle 1| \hat{H}_1, \quad (3)$$

$$\hat{H}_0 = \hat{H}_{n-n} + \sum_{i=1}^{n} \omega_0 \hat{I}_z^i, \quad (4)$$

$$\hat{H}_1 = \hat{H}_{n-n} + \sum_{i=1}^{n} (\omega_0 - A_{\parallel}^i) \hat{I}_z^i + A_{\perp}^i \hat{I}_x^i, \quad (5)$$

where H_0 (H_1) is the Hamiltonian describing the rest of the system if the electron is in the state $m_s = 0$ $(m_s = -1)$.

Supplementary Note 2: Pseudo-spin model

Under high magnetic field, the dynamics of a $^{13}C - ^{13}C$ pair can be approximated by a pseudo-spin-$\frac{1}{2}$ model [1, 2], where the two anti-parallel spin states of the pair ($|\uparrow\downarrow\rangle$ and $|\downarrow\uparrow\rangle$) are mapped into spin-up ($|\Uparrow\rangle$) and spin-down ($|\Downarrow\rangle$) states of the pseudo-spin. The polarized states ($|\uparrow\uparrow\rangle$ and $|\downarrow\downarrow\rangle$) have large energy separation (due to large Zeeman energy) with respect to other states and thus do not play a role in the dynamics. Under these assumptions, the dynamics of the pseudo-spin can be described conditional on the electron spin state by the Hamiltonian:

$$\hat{H}_0 = X\hat{S}_x, \text{ and } \hat{H}_1 = X\hat{S}_x + Z\hat{S}_z, \quad (6)$$

where $\hat{H}_0$ ($\hat{H}_1$) is the Hamiltonian if the electron is in $m_s = 0$ ($m_s = -1$), X is the dipolar coupling strength between the two nuclear spins [3]:

$$X = \frac{\mu_0}{4\pi}\frac{\gamma_c^2}{r^3}\frac{1}{2}(1 - 3\cos^2\theta), \tag{7}$$

where γ_c is the gyromagnetic ratio of the ^{13}C nuclear spin, r is the distance between the two nuclear spins forming the pair, θ is the angle between the pair axis $\mathbf{r}$ and the external magnetic field direction ([1,1,1] in our case). Z is due to the hyperfine field gradient [2],

$$Z = Z_{\parallel} + Z_{\perp} = (A_{\parallel}^1 - A_{\parallel}^2) + \frac{(A_{\perp}^1)^2 - (A_{\perp}^2)^2}{2\pi \cdot \gamma_C B_0}. \tag{8}$$

Supplementary Table 3 shows the calculated set of possible coupling strengths for pairs with different distances, r, and angles, θ, starting from the most strongly coupled pair (2.061 kHz) down to a coupling strength of 61 Hz. This is the range of interest for the pairs that we detect in this work. The values of the coupling strength X are distinct for different possible pairs and thus enable us to determine the distance between the two nuclear spins forming the pair and their orientation with respect to the external field. An exception is pair 3, for which two different types of pairs yield values close to the experimental value (see Supplementary table 4). Although this information is enough to describe the dynamics under dynamical decoupling and the electron spin coherence, the measured value of Z for a single electron-spin state, i.e. $m_s = -1$ in our case, does not yet enable us to uniquely determine the relative position of the pair with respect to the NV. Measuring Z for $m_s = +1$ as well enables obtaining the two quantities $(A_{\parallel}^1 - A_{\parallel}^2)$ and $(A_{\perp}^1)^2 - (A_{\perp}^2)^2$, which would further narrow down the possible pair positions [2].

Supplementary Note 3: Effect of magnetic field misalignment on ^{13}C precession frequencies

A misaligned field from the NV-axis would give rise to non-secular terms in the Hamiltonian leading to an effective g-tensor for ^{13}C nuclear spins that depends on the hyperfine coupling strength between the electron and the ^{13}C nuclear spin. For the electron in $m_s = 0$, this effective g-tensor can be calculated as follows [4]:

$$\mathbf{g}(m_s = 0) = \begin{bmatrix} 1 + \eta A_{xx} & \eta A_{xy} & \eta A_{xz} \\ \eta A_{xy} & 1 + \eta A_{yy} & \eta A_{yz} \\ 0 & 0 & 1 \end{bmatrix}, \tag{9}$$

where $\eta = \frac{2\gamma_e}{2\pi\cdot\gamma_c\Delta} = \frac{1}{2\pi}\cdot 1.824\cdot 10^{-3}$ kHz^{-1}, and A_{mn} is the hyperfine tensor between the electron and ^{13}C nuclear spin. The ^{13}C precession frequency can now be calculated as $\omega_0 = |2\pi\gamma_c\mathbf{B}\cdot\mathbf{g}(0)|$ [4]. We estimate our magnetic field alignment to be better than 0.35 degrees, which corresponds to a maximum perpendicular field component of 2.5 G (see Supplementary Table 1). Now if we assume that our field lies in xz-plane, i.e. $\mathbf{B} = B_z\mathbf{e_z} + B_x\mathbf{e_x}$, then $\mathbf{B}\cdot\mathbf{g} = B_x(1+\eta A_{xx})\mathbf{e_x} + \eta B_x A_{xy}\mathbf{e_y} + (\eta B_x A_{xz} + B_z)\mathbf{e_z}$, which leads to :

$$\omega_0 = 2\pi\cdot\gamma_c\sqrt{[B_x(1+\eta A_{xx})]^2 + [\eta B_x A_{xy}]^2 + [B_z + \eta B_x A_{xz}]^2} \quad (10)$$

In our case A_{xz} and A_{xx} range from $-2\pi\cdot 50$ to $2\pi\cdot 50$ kHz (at maximum), and the maximum value of B_x is 2.5 G. This means that different nuclear spins would have different precession frequencies, ω_0, depending on their hyperfine coupling parameters. The dominant term of change in ω_0 with the hyperfine coupling strengths, for our range of parameters, is $2\pi\cdot\gamma_c\eta B_x A_{xz}$, which would lead to a maximum difference in ω_0 of $2\pi\cdot 500$ Hz between different nuclear spins. This is consistent with what we experimentally observe (see Supplementary Table 2).

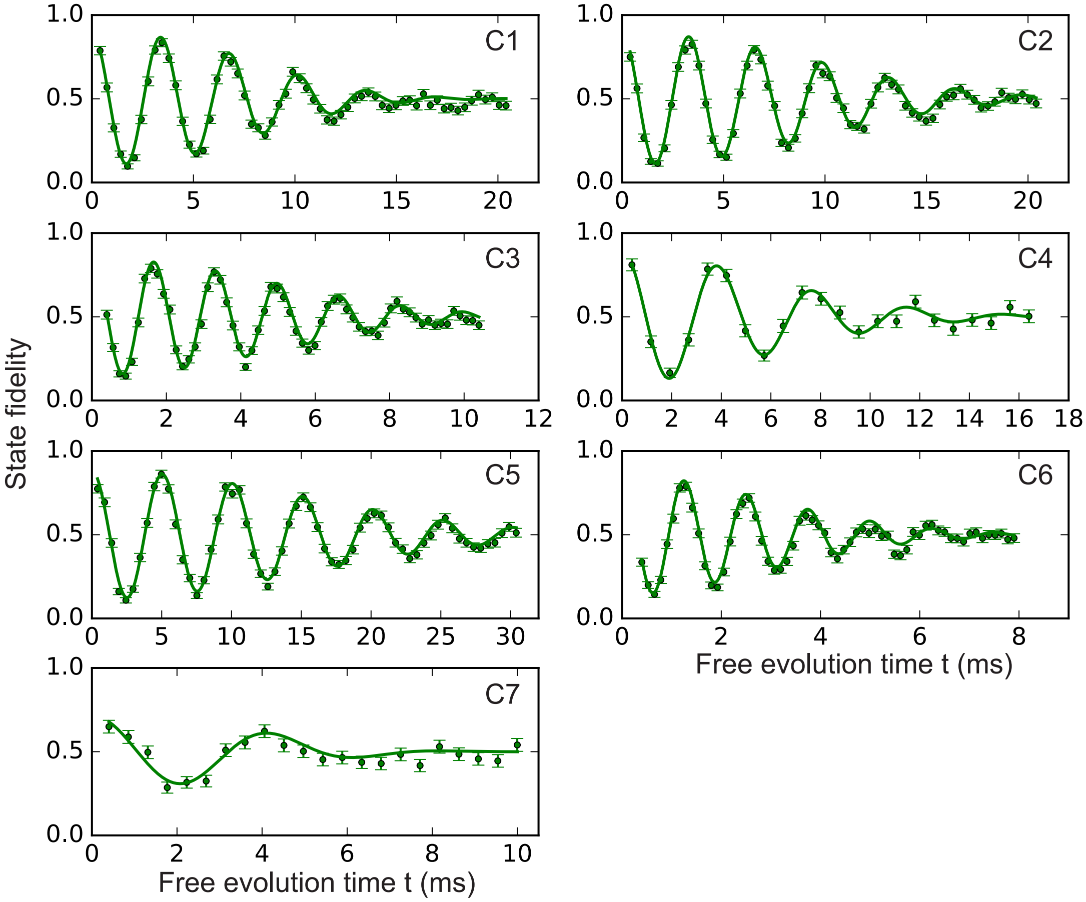

Supplementary Figure 1. **Ramsey experiments for the seven individual ^{13}C spins.** Ramsey interferometry [5] for the ^{13}C nuclear spins. The electron spin state during the free evolution time is $m_s = -1$ (+1 for C4 and C7). Lines are sinusoidal fits with a Gaussian decay: $F = a + A \cdot e^{-(t/T_2^*)^2} \cos(\delta t + \phi)$, with t the free evolution time and δ a detuning. All seven spin signals are well described by a single, unique precession frequency $\omega_{\pm 1} \approx \omega_0 \pm A_{\parallel}$ (see Supplementary Table 2) and a Gaussian decay, indicating that all seven spins are distinct and that none couple strongly to other ^{13}C spins in the environment. The minimum coupling strength for the observed ^{13}C-^{13}C pairs of 83 Hz (Supplementary Table 4), would already introduce a clear beating in ~ 3 ms, indicating that the seven identified single ^{13}C spins are not part of the 6 detected ^{13}C - ^{13}C pairs. All error bars are one statistical s.d.

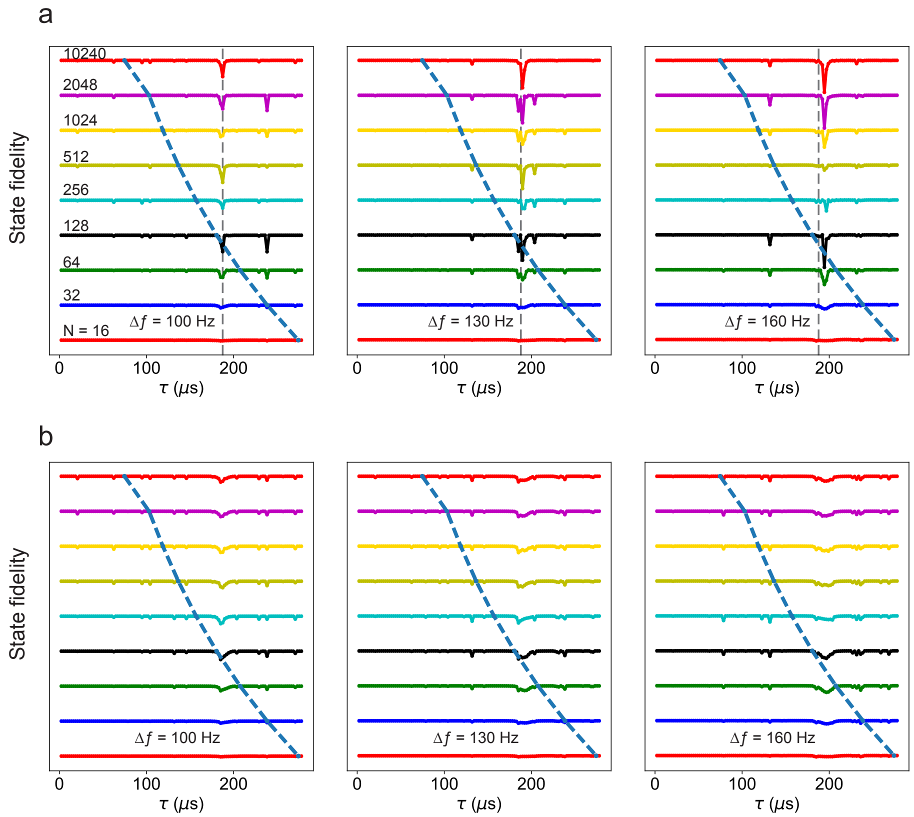

Supplementary Figure 2. **Effect of deviations of τ from** $2\pi/\omega_0$**.** To avoid coupling to the single ^{13}C spins, we aim to set the interpulse spacing to the revival condition $\tau = m \cdot 2\pi/\omega_0$, with m an integer and ω_0 the ^{13}C frequency for $m_s = 0$ [4]. However, this condition is not met exactly and simultaneously in the experiments for all ^{13}C spins (Supplementary Note 3 and Supplementary Table 2). Here we explore the effect of small deviations from this condition. **a)** Simulated electron spin fidelity after a decoupling sequence with $\tau = m \cdot \frac{2\pi}{\omega_L}$, with ω_L the ^{13}C Larmor frequency estimated from ESR measurements (Supplementary Table 1). In these simulations we include the seven characterized ^{13}C spins and set all precession frequencies to $\omega_0 = \omega_L - \Delta\omega$. The curves show results for $\frac{\Delta\omega}{2\pi} = 100, 130$ and 160 Hz. The y-axis scale is such that the difference between horizontal lines at $\tau = 0$ is 1. The dashed blue line marks the $1/e$ decay times for different values of N (from Fig. 5); the main region of interest lies to the left of this line. Note that since the coherence time does not scale linearly with the number of pulses but rather with $N^{0.799}$, the $1/e$ value for τ becomes shorter for larger N. The vertical gray dashed line provides a visual aid to illustrate how the dip positions change with $\Delta\omega$. This shows that a change of 30 Hz in $\frac{\Delta\omega}{2\pi}$ leads to variations of the dip pattern. **b)** The obtained state fidelity averaged over 500 repetitions with ω_0 for the seven spins drawn from a Gaussian distribution with a mean frequency of $\omega_L - \Delta\omega$ and standard deviation of 30 Hz. These fluctuations match the typical observed values of $T_2{}^*$ for the nuclear spins. The result shows that differences in $m_s = 0$ frequencies for different ^{13}C spins are smeared out by dephasing, so that their net effect on the decoupling curves is small. Additionally, the interpulse delay is set with a precision of $\delta_p = 1$ ns. The maximum relative error occurs at short τ ($\tau = \tau_L$) and is of order $\delta_p/2\tau$. This is equivalent to a $\Delta\omega \sim 2\pi \cdot 100$ Hz, for which the simulations show a negligible effect at short τ. At larger τ ($\tau > 10\tau_L$) the relative error in τ quickly becomes negligible.

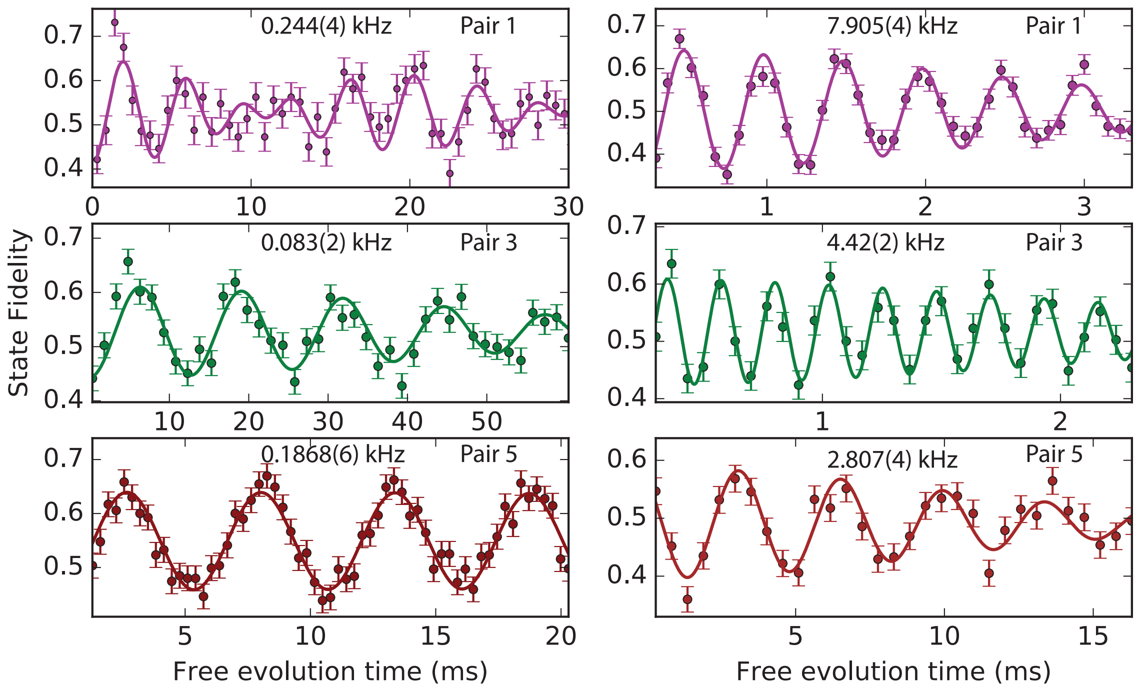

Supplementary Figure 3. **Direct spectroscopy of ^{13}C-^{13}C pairs.** Ramsey spectroscopy for pairs 1, 3 and 5 and for electron state $m_s = 0$ (left) and $m_s = -1$ (right) during the free evolution time. The measurement sequence is shown in Fig. 3a. These pairs are all of the type $Z >> X$. For the measurements with $m_s = -1$ an artificial detuning was applied. Pair 1 shows an additional beating (frequency of 22(2) Hz) indicating a small coupling to one (or more) additional spins. Parameters and fit results are summarized in Supplementary Table 4. All error bars are one statistical s.d.

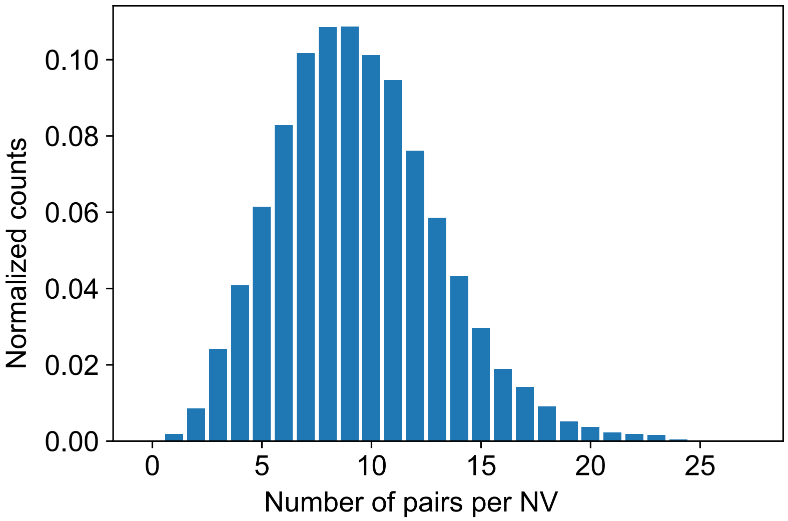

Supplementary Figure 4. Statistics for the number of ^{13}C pairs per NV center with coupling parameters in the range of the experimentally observed pairs. To estimate if the observed number of pairs is consistent with the ^{13}C concentration (1.1%), we generate 10000 NV centers with random nuclear spin baths. For each NV, all pairs with coupling strength in the range $X = 2\pi \cdot 75$ Hz to $X = 2\pi \cdot 2061$ Hz are identified. For the range $2\pi \cdot 75 \text{ Hz} \leqslant X \leqslant 2\pi \cdot 687$ Hz, we count the pairs for which the first resonance lies within our experimental window: $\tau_r = \frac{\pi}{2\omega_r} < 300\ \mu$s, with $\omega_r = \sqrt{X^2 + (Z/2)^2}$. This lead to the condition: $\sqrt{X^2 + (Z/2)^2} > 2\pi \cdot 833$ Hz. For pairs with $X = 2\pi \cdot 2.061$ kHz the signal always lies within the 300 μs window, however for the signal strength to be significant Z must be sufficiently large. We count all such pairs with $Z > 50$ Hz. The resulting statistics for the total number of pairs per NV gives an average of 9.4 with a standard deviation of 3.7. Although this estimate doesn't take into account all the subtleties of the possible dynamics of the pairs, it indicates that the number of observed pairs is consistent with the ^{13}C concentration.

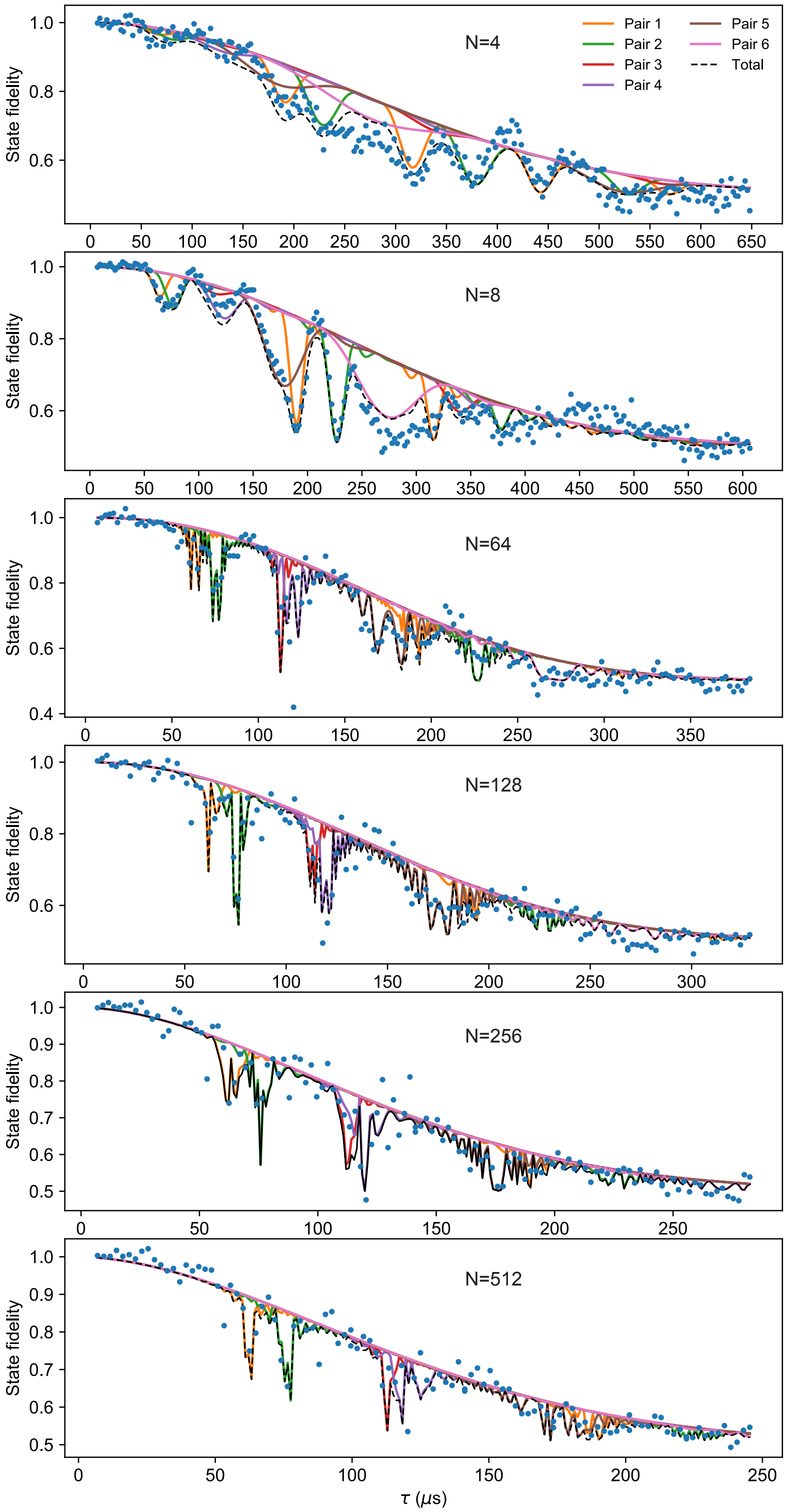

Supplementary Figure 5. **Comparison of the calculated signal for the six $^{13}C - ^{13}C$ pairs to the decoupling data.** Similar to the examples in Fig. 4 for N = 16 and 32, here we show extra examples for different N to confirm that the six identified $^{13}C - ^{13}C$ pairs provide a good description of the dynamical decoupling data. $\tau = m \cdot \frac{2\pi}{\omega_L}$ to avoid effects of coupling to individual ^{13}C spins.

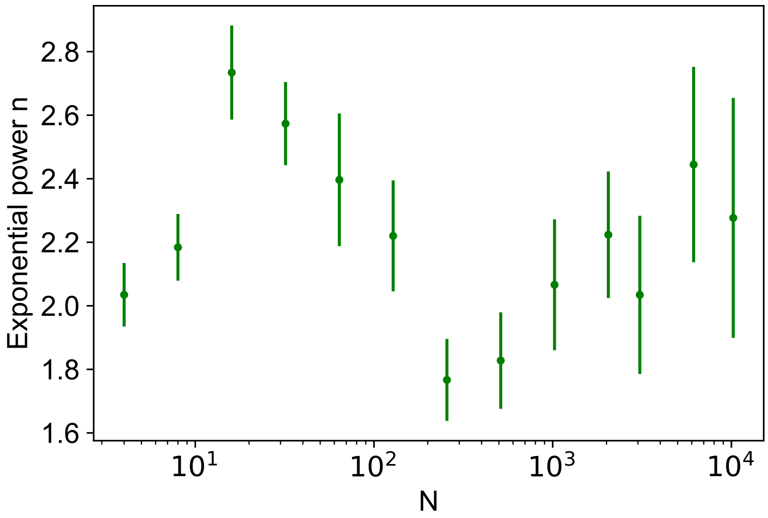

Supplementary Figure 6. **Extracted exponent of the coherence decay.** Fitted values of n for the $e^{-(\tau/T)^n}$ envelop decay for the different numbers of pulses N in Fig. 5a. The fact that the value is around 2 even for $N = 10^4$ pulses confirms that coherence times are not yet limited by T_1 (expected $n = 1$ for T_1-limited case). All error bars are one statistical s.d.

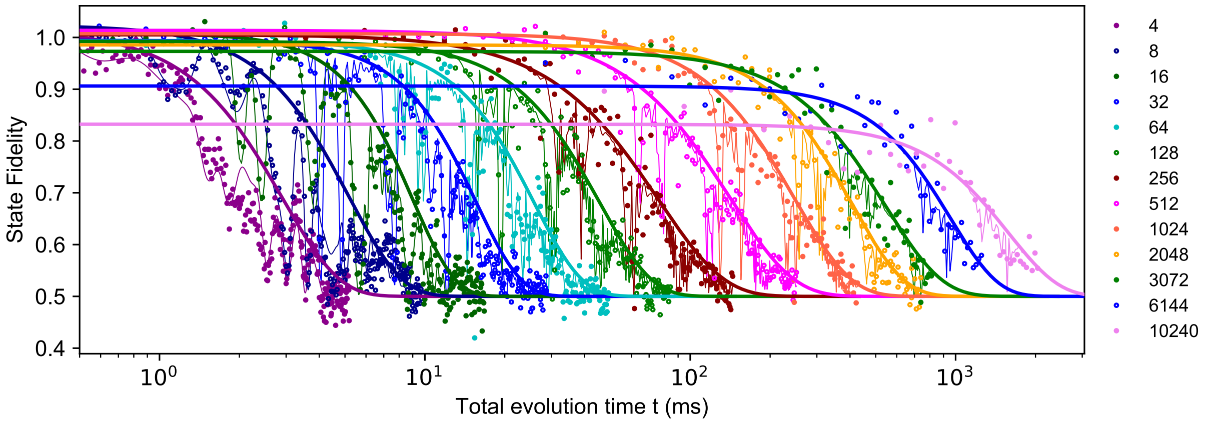

Supplementary Figure 7. Dynamical decoupling data of Fig. 5a without normalization. Measured state fidelities under dynamical decoupling with the number of pulses varying from $N = 4$ to $N = 10240$. Pulse errors become significant for higher number of pulses causing a decrease in the initial amplitude A (see also Supplementary Fig. 8).

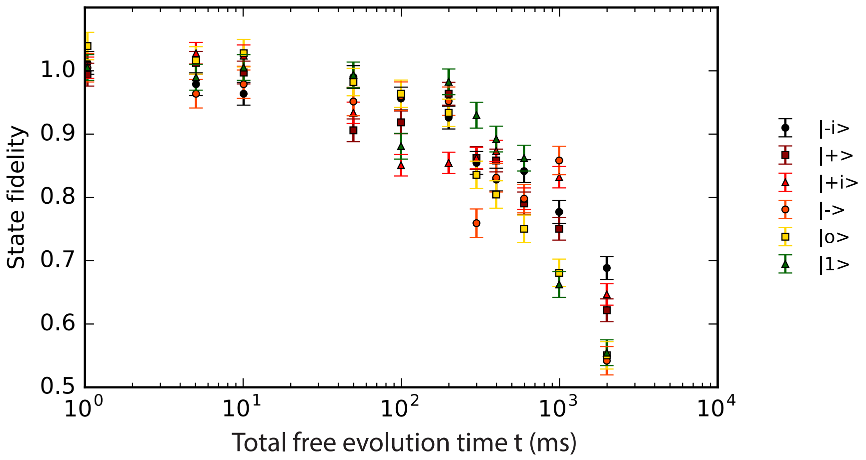

Supplementary Figure 8. **State fidelity for the six states used in Fig. 5c.** We prepare the six states: $|0\rangle = |m_s = 0\rangle$, $|1\rangle = |m_s = -1\rangle$, $|\pm\rangle = (|0\rangle \pm |1\rangle)$, $| \pm i\rangle = (|0\rangle \pm i|1\rangle)$ and measure the fidelity of the final state with the ideal initial state. The curve in Fig. 5c in the main text is the average of these six state fidelities. The spin eigenstates $|0\rangle$ and $|1\rangle$ show a similar decay to the superposition states, indicating that the fidelities are likely limited by pulse errors. All error bars are one statistical s.d.

$m_s = -1$ transition (f_{-1})	1.746666(3) GHz
$m_s = +1$ transition (f_{+1})	4.008580(3) GHz
Zero field splitting (Δ)	2.877623 GHz
Magnetic field B_z	403.553 G
Magnetic field stability	$3 \cdot 10^{-3}$ G
Magnetic field alignment	< 0.35 degrees
Electron T_2^*	4.9(2) μs
Electron T_2	1.182(5) ms
Electron T_1	$3.6(3) \cdot 10^3$ s
Frequency for pulse spacing $\omega_L/2\pi$	432.004 kHz
Period for pulse spacing τ_L	2.3147 μs
Electron Rabi freq.	14.31(3) MHz
NV strain	4.0 GHz

Supplementary Table 1. **Experimental parameters.** $m_s = -1$ and $m_s = +1$ transitions are the obtained frequencies from electron spin resonance (ESR) measurements for the two spin transitions $0 \to -1$ and $0 \to +1$. Assuming a well-aligned field with the NV axis, the zero field splitting (ZFS) is the average of the two frequencies. The magnetic field B_z is the estimated field strength from $m_s = -1$ and $m_s = +1$ frequencies ($B_z = \frac{f_{+1} - f_{-1}}{2\gamma_e}, \gamma_e = 2.8024$ MHz G^{-1}). The magnetic field stability is the standard deviation of the magnetic field measured continuously (typical measurement time is 80 s) for 5 hours, during which the magnetic field is re-calibrated every 30 minutes, just as during the actual measurements. The magnetic field is aligned with the NV axis by sweeping the magnet position in the transversal directions and minimizing $\frac{f_{+1}+f_{-1}}{2}$. The maximum deviation from the minimum is estimated to be 10 kHz. This implies a maximum perpendicular field $B_\perp$ of 2.5 G, equivalent to a maximum misalignment angle of 0.35 degrees. Electron T_2^* is the free induction decay of the electron spin measured in a Ramsey interference experiment. Electron T_2 is the 1/e decay time of a spin echo measurement. Electron T_1 is the electron relaxation time shown in Fig. 1. $\omega_L/2\pi$ is the ^{13}C Larmor frequency for $m_s = 0$ estimated from the ESR measurements ($\omega_L = 2\pi\gamma_c \cdot B_z$, $\gamma_c = 1.0705$ kHz G^{-1}). τ_L is the estimated ^{13}C Larmor period ($\frac{2\pi}{\omega_L}$) as used for setting the half interpulse delay ($\tau = m \cdot \tau_L$) in the dynamical decoupling sequences. NV strain is the splitting of the excited states E_x and E_y due to strain perpendicular to the NV axis, measured by a resonant excitation spectroscopy at a temperature of 3.7 K.

	$\omega_0/2\pi$ (kHz)	$\omega_1/2\pi$ (kHz)	T_2^* (ms)	$A_\parallel/2\pi$ (kHz)	$A_\perp/2\pi$(kHz)
C1	431.994(3)	469.320(5)	10.2(4)	-36.4	25
C2	431.874(3)	413.739(1)	12.5(5)	20.6	43
C3	431.891(2)	447.209(2)	6.6(3)	-11.4	59
C4	431.947(3)	440.740(2)	8.3 (6)	8.1	21
C5	431.934(3)	408.303(3)	20.8(7)	24.4	26
C6	431.960(1)	480.607(4)	4.0(2)	-48.7	12
C7	431.95(1)	446.63(1)	5.0(7)	14.5	11

Supplementary Table 2. **Spectroscopy of isolated** ^{13}C**.** ω_0 and ω_1 are the measured nuclear precession frequencies for $m_s = 0$ and $m_s = -1$ ($m_s = +1$ for C4 and C7), obtained from least-squares fits of the Ramsey signals (e.g. Supplementary Fig. 1) to sinusoidal functions with Gaussian decays. T_2^* is the dephasing time for $m_s = -1$ ($m_s = +1$ for C4 and C7) obtained from the same fit. We use the variation in ω_0 for these spins as an estimate of how much ω_0 varies between spins. This variation can be explained by an effective g-tensor for ^{13}C nuclear spins, due to a slightly misaligned field (see Supplementary Note 3). We study how these variations can affect the dynamical decoupling signal in Supplementary Fig. 2. $A_\parallel$ and $A_\perp$ are estimates for the hyperfine interaction components parallel and perpendicular to the applied magnetic field, obtained by matching the theoretical signal (e.g. Fig. 2a, b) to the observed electron spin coherence. Uncertainties are estimated to be of the order of the last digit.

**r**	r	θ (degrees)	$\lvert X\rvert/2\pi$ (Hz)
$\frac{a_0}{4}$[1,1,1]	$0.433a_0$	0	2061.0
$\frac{a_0}{4}$[1,-1,1]	$0.433a_0$	70.5	687.0
$\frac{a_0}{4}$[±2,2,0]	$0.707a_0$	35.3/90	236.7
$\frac{a_0}{4}$[1,1,3]	$0.829a_0$	29.5	186.8
$\frac{a_0}{4}$[1,-3,1]	$0.829a_0$	80.0	133.4
$\frac{a_0}{4}$[3,1,3]	$1.089a_0$	22.0	102.1
$\frac{a_0}{4}$[3,3,3]	$1.299a_0$	0	76.3
$\frac{a_0}{4}$[2,2,4]	$1.225a_0$	19.5	75.9
$\frac{a_0}{4}$[3,-1,-3]	$1.089a_0$	82.4	61.3

Supplementary Table 3. **Main $^{13}C-^{13}C$ pairs in the diamond lattice and their calculated coupling strengths.** The coupling strength X is given by equation (7). **r** is the vector connecting the $^{13}C-^{13}C$ pair; θ is the angle between the pair axis and the external magnetic field which is along [1,1,1]. The distance between the two carbons forming the pair is r. The diamond lattice constant is taken to be $a_0 = 3.5668$ Å at 3.7 K [6]. This table shows pairs with coupling strength down to 61 Hz which covers the range of pairs that we observed in this work. For the pairs with $X = 236.7$ Hz two different angles with respect to the magnetic field are possible.

	τ (μs)	N	$\omega_0/2\pi$ (kHz)	$X_{theory}/2\pi$ (kHz)	$\omega_{-1}/2\pi$ (kHz)	$Z/2\pi$ (kHz)
Pair 1	63	14	0.244(3)	0.2367	7.894(9)	7.890(9)
Pair 2	76	10	0.247(6)	0.2367	6.587(7)	6.582(6)
Pair 3	111	26	0.083(2)	0.0759/0.0763	4.42(2)	4.42(2)
Pair 4	120	24	2.0827(7)	2.061	2.0843(2)	0.230
Pair 5	172	8	0.1868(6)	0.1868	2.807(4)	2.801(4)
Pair 6	277	8	0.1338(1)	0.1334	1.831(3)	1.826(3)

Supplementary Table 4. **Parameters for the six $^{13}C - ^{13}C$ pairs.** τ is half of the interpulse delay and N is the number of pulses in the decoupling sequence used to perform the conditional gates in the Ramsey measurement sequences shown in Fig. 3. ω_0 and ω_{-1} are the measured pseudo-spin precession frequencies for $m_s = 0$ and $m_s = -1$ respectively. $\omega_0/2\pi$ is a direct measurement of the coupling strength X and X_{theory} is the closest theoretical dipolar coupling strength to this value. This can be used to determine the atomic structure of the pair as shown in Supplementary Table 3. Z is due to the hyperfine field gradient and is calculated from the measured ω_0 and ω_{-1}: $Z = \sqrt{\omega_{-1}^2 - \omega_0^2}$. Note that for pair 4 we have $X >> Z$, so the resonance condition is mainly governed by the coupling strength X (resonant $\tau \sim 120$ μs). Therefore, it is likely that additional pairs with the same X — but smaller Z values — contribute to the observed signal at 120 μs. Here we match the measured dynamical decoupling data for different values of N (see e.g. Fig. 4b) to the calculated signal for a single pair, and find that the results are accurately reproduced for $Z/2\pi = 0.230$ kHz. Note that for pair 3, there are two possible configurations with theoretical coupling strength close to the measured value.

Supplementary References

[1] Zhao, N. *et al.* Sensing single remote nuclear spins. *Nat. Nanotech.* **7**, 657–662 (2012).

[2] Shi, F. *et al.* Sensing and atomic-scale structure analysis of single nuclear-spin clusters in diamond. *Nat. Phys.* **10**, 21–25 (2014).

[3] Zhao, N., Hu, J.-L., Ho, S.-W., Wan, J. T. K. & Liu, R. B. Atomic-scale magnetometry of distant nuclear spin clusters via nitrogen-vacancy spin in diamond. *Nat. Nanotech.* **6**, 242–246 (2011).

[4] Childress, L. *et al.* Coherent dynamics of coupled electron and nuclear spin qubits in diamond. *Science* **314**, 281–285 (2006).

[5] Cramer, J. *et al.* Repeated quantum error correction on a continuously encoded qubit by real-time feedback. *Nat. Commun.* **7**, 11526 (2016).

[6] Stoupin, S. & Shvyd'ko, Y. V. Thermal expansion of diamond at low temperatures. *Phys. Rev. Lett.* **104**, 085901 (2010).
